# Telemedicine – a scientometric and density equalizing analysis

**DOI:** 10.1186/s12995-015-0076-3

**Published:** 2015-10-24

**Authors:** David A. Groneberg, Shaghayegh Rahimian, Matthias Bundschuh, Mario Schwarzer, Alexander Gerber, Beatrix Kloft

**Affiliations:** Department of Health Economics, The Institute of Occupational Medicine, Social Medicine and Environmental Medicine, Goethe University Frankfurt, Theodor-Stern-Kai 7, 60590 Frankfurt, Germany; Institute of Occupational Medicine, Charité School of Medicine, Thielallee 73, 14195 Berlin, Germany; Health Economics, Department of Gynecology and Obstetrics, Goethe University Frankfurt, Theodor-Stern-Kai 7, 60590 Frankfurt, Germany

**Keywords:** Telemedicine, Bibliometry, Scientometry, Density equalizing map, Citation

## Abstract

**Background:**

As a result of the various telemedicine projects in the past years a large number of studies were recently published in this field. However, a precise bibliometric analysis of telemedicine publications does not exist so far.

**Methods:**

The present study was conducted to establish a data base of the existing approaches. Density-equalizing algorithms were used and data was retrieved from the Thomson Reuters database Web of Science.

**Results:**

During the period from 1900 to 2006 a number of 3290 filed items were connected to telemedicine, with the first being published in 1964. The studies originate from 101 countries, with the USA, Great Britain and Canada being the most productive suppliers participating in 56.08 % of all published items. Analyzing the average citation per item for countries with more than 10 publications, Ireland ranked first (10.19/item), New Zealand ranked second (9.5/item) followed by Finland (9.04/item). The citation rate can be assumed as an indicator for research quality. The ten most productive journals include three journals with the main focus telemedicine and another five with the main focus “Information/Informatics”. In all subject categories examined for published items related to telemedicine, “Health Care Sciences & Services” ranked first by far. More than 36 % of all publications are assigned to this category, followed by “Medical Informatics” with 9.72 % and “Medicine, General & Internal” with 8.84 % of all publications.

**Conclusion:**

In summary it can be concluded that the data shows clearly a strong increase in research productivity. Using science citation analysis it can be assumed that there is a large rise in the interest in telemedicine studies.

## Introduction

Telemedicine and telemetrics are increasingly important for occupational and environmental medicine and many routine and research approaches use technology of this field for different purposes [1]. One definition is that “Telemedicine is the use of electronic information and communication technologies to provide and support health care when distance separates the participants” [2]. The first telemedical activities were documented in 1897, when the telephone was used to confirm a diagnosis [3], and there are reports from 1922 which certify the exchange of medical information between ships and coast guards [4]. Since then telemedicine plays an important role for all aspects of public health. Especially in areas, which are not densely populated, telemedicine may serve as a powerful tool to implement improvements of the public health system.

The invention of television induced the first wave of telemedicine projects. In 1959 Jutras succeeded to interconnect several hospitals via a cable supported TV system. This system enabled the transmission of roentgenograms over a distance of up to five miles [5]. With the development of new telecommunication technologies such as UMTS, infrared or Bluetooth telemedicine is today a growth market with high potentials and great opportunities for global health care. Telemedicine is most beneficial for populations living in isolated communities and remote regions and is currently being applied in virtually all medical domains. This new technology has mostly been used as a communication and consultation tool between a general practitioner and a specialist available at a remote location. In recent years, the focus has also been on monitoring a patient at home using known devices like blood pressure monitors and transferring the information to a caregiver. These telemonitoring solutions have a focus on current high morbidity chronic diseases, such as chronic heart failure or diabetes mellitus. Telemedicine holds big promises to solving major health care problems; hence, in the last years various studies were concluded. However, there is no in-depth bibliometric analysis of the current state of research regarding telemedicine available. Due to the importance of telemedicine for public health, the NewQIS project (New Quality and Quantity Indices in Science) [6–8] elected the topic of telemedicine as a primary research focus and established an in depth scientometric study. Large-scale data analysis and bibliometric approaches including density-equalizing mapping were performed.

## Methods

### Data source

The project is embedded in the NewQIS platform which already addressed numerous topics with importance to the field of public health Data [6, 9] was retrieved from the database Web of Science from the former Institute for Scientific Information of Thomson Reuters [10].

### Search strategies

For the different searches the keyword telemedicine was used. The search included all document types (e.g. original articles, reviews, letters, editorials and news reports).

### Time span

The initially analyzed time span included the period from 1900 to 2006. The last seven years were not analyzed since the evaluability of the number of citations is not given and it hardly allows to draw conclusions. Thus, the half live of citations has confined the period of investigation so far. To examine particular aspects of the retrieved data in depth, the time span was partly restricted to a period between 1976 and 2006.

### Citation quantities

Published items were further analyzed [6, 11, 12] by using the function “citation report” of the Web of Science database. This method was used to examine the number of citations per citation year, the number of citations per publishing year and the average number of citations per item.

### Data categorization

In accordance with the NewQIS platform [6, 9, 13], all data files were analyzed regarding a variety of different aspects, e.g. the publishing countries and languages, the publication years, the source titles and subject categories. Subsequently, the data was transferred to charts, analyzed and the findings visualized.

Density-equalizing mapping was used according to a method of Gastner and Newman [14]. With the application of this technique, territories were resized depending on a particular variable, e.g. the number of published items. For the resizing procedure the area of each country was scaled in proportion to its total number of published items regarding telemedicine.

## Results

### Total number of published items

**Fig. 1:**
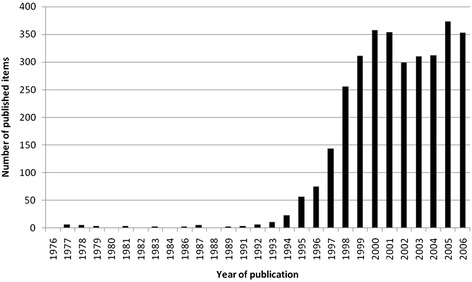
Published items related to telemedicine in the Web of Science database 1900–2006

The number of published items was used as an indication of research productivity. During the period 1900–2006 3290 items with the keyword telemedicine were identified. The first studies were published in the year 1964, but till the early 1990ies the total quantity of publications was insignificant. However, numbers increase highly in the middle of the 1990ies. The number of published items jumps from 23 in the year 1994 up to 143 in 1997 and 357 in the year 2000. This is a considerable increase of 1450 % in a period of 6 years (Fig. 1).

### Analysis of origin

**Fig. 2:**
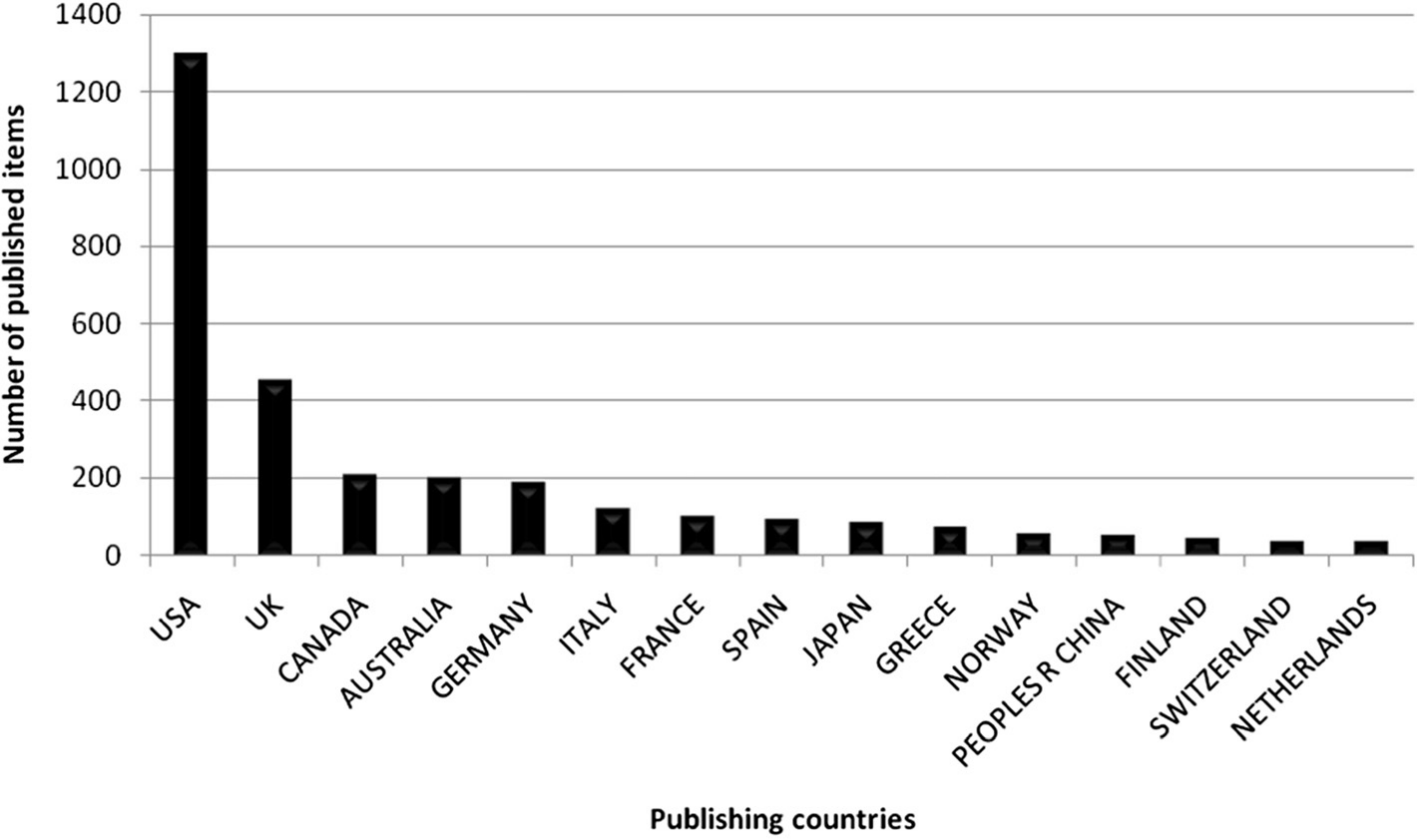
Ranking of country total numbers of published items related to telemedicine

**Fig. 3:**
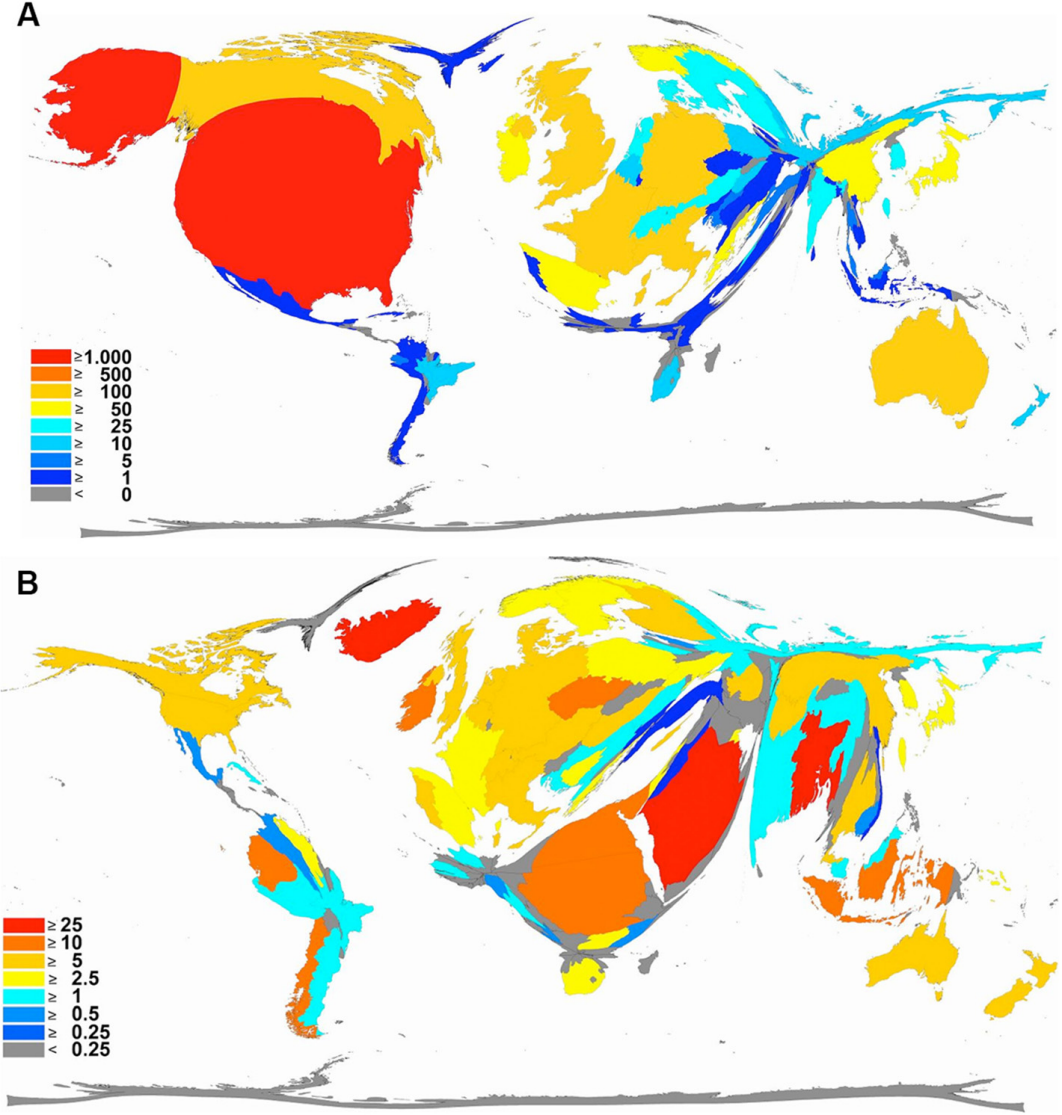
**a** Density-equalizing map illustrating the number of publications in each particular country. The area of each country was scaled in proportion to its total number of publications regarding telemedicine. **b** Density-equalizing map showing the average citations per item of each particular country. The area of each country was scaled in proportion to its average number of citations per item regarding telemedicine

The 3290 identified publications were originated from 101 countries, with the USA, Great Britain and Canada being the most productive ones (Fig. 2) participating in 56.08 % of all published items. Australia and Germany are also among the top five countries with the highest number of studies regarding telemedicine. The positions five to ten go to Italy, France, Spain, Japan and Greece. The cumulated publications of the top ten publishing countries encompassed 82.26 % of all published items. Density-equalizing mapping of this set of data demonstrates that a relatively small number of countries is responsible for the majority of research efforts (Fig. 3a).

### Citation parameters

**Fig. 4:**
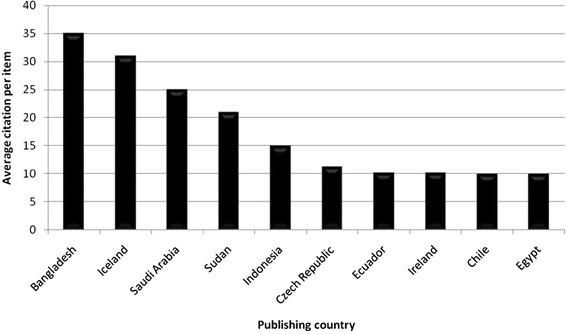
Average citation per item of each particular country

**Fig. 5:**
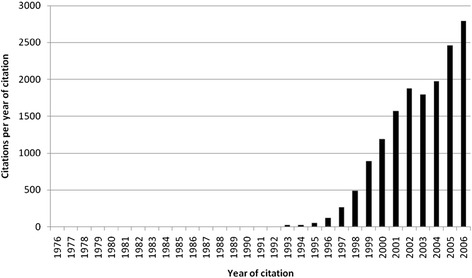
Citations per year of citation

**Fig. 6:**
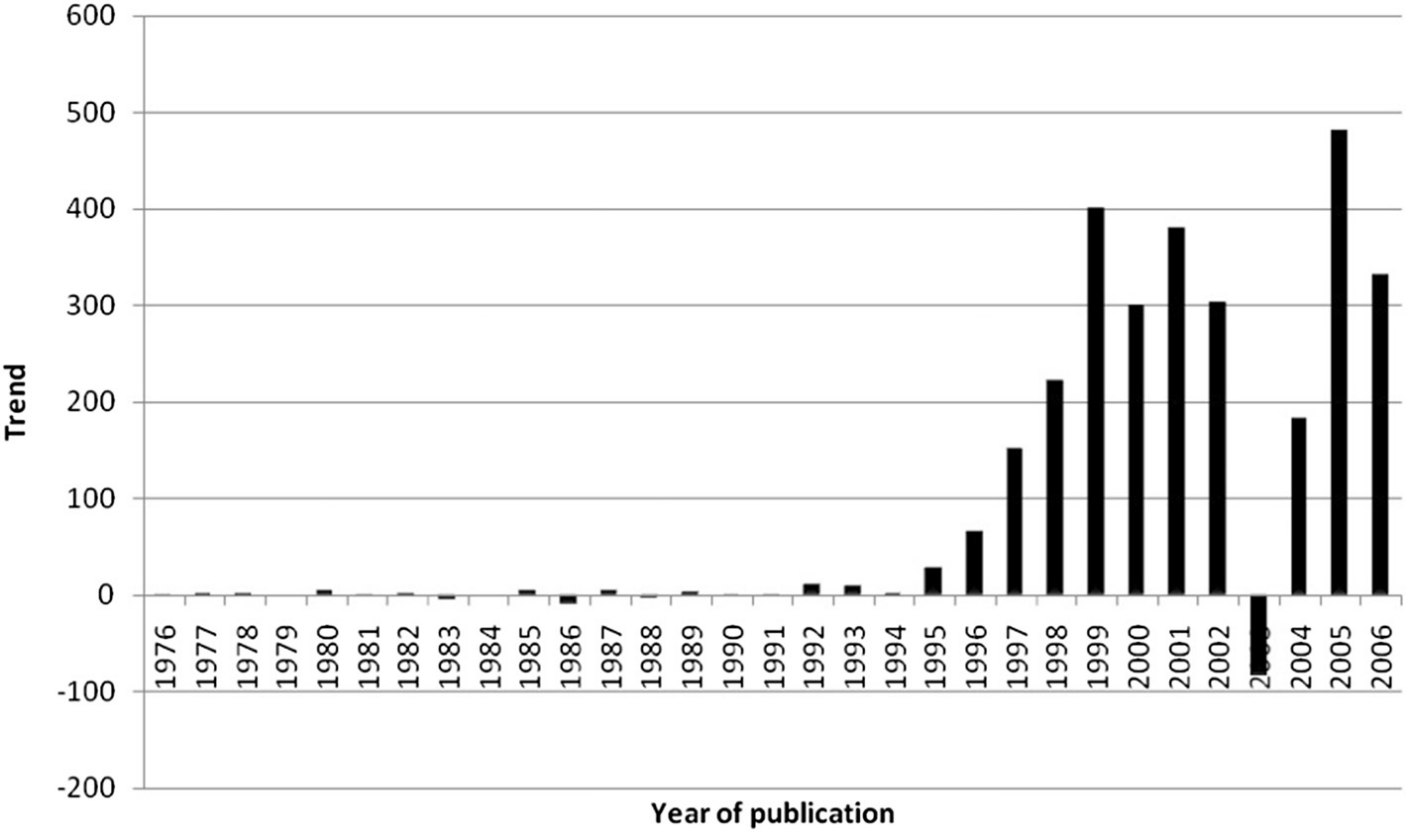
Increase and decrease of citations per year of citation

The average citation per item was used as an indicator for research quality, and differences were found in relation to research quantity figures: when analyzing all 3290 published items regarding the average citation of each item in a country-specific manner, Bangladesh has the highest average citation rate (35/item), with Iceland ranking second (31/item) and Saudi Arabia ranking third (25/item) (Fig. 4, Table 1). Differences to output quantity (Fig. 3a) can be visualized by the use of a density-equalizing calculation to provide a global scheme of the average citations per item of each country (Fig. 3b). When a threshold of at least ten publications is set up, Bangladesh (1 publication), Iceland (1 publication) and Saudi Arabia (1 publication) are no longer ranked in the top ten, and Ireland (80 publications) moves into first position, with an average citation rate of 10.19/item. Additionally New Zealand with 9.5 citations per item and Finland with 9.04 citations per item enter the top three (Table 2). To assess the reception of the subject matter over the time, the number of citations per citation year was recorded from 1976 to 2006 (Fig. 5) and a trend of increasing citations was present since 1976 (Fig. 6), which parallels the increase in published articles.

**Table 1:** Average citation per item of each particular country and total number of publications

Country	Average citation per Item	Number of publications
Bangladesh	35	1
Iceland	31	1
Saudi Arabia	25	1
Sudan	21	1
Indonesia	15	1
Czech Republic	11.25	4
Ecuador	10.2	5
Ireland	10.19	80
Chile	10	3
Egypt	10	1

**Table 2:** Average citation per item of each particular country and the total number of publications (threshold excludes countries with < 10 publications)

Country	Average citation per Item	Number of publications
Ireland	10.19	80
New Zealand	9.5	12
Finland	9.04	46
Denmark	8.72	25
USA	7.41	1,297
Austria	7.26	34
Belgium	7.24	17
China	7.05	56
Switzerland	6.87	39
Italy	6.72	120

### Publishing journals

**Fig. 7:**
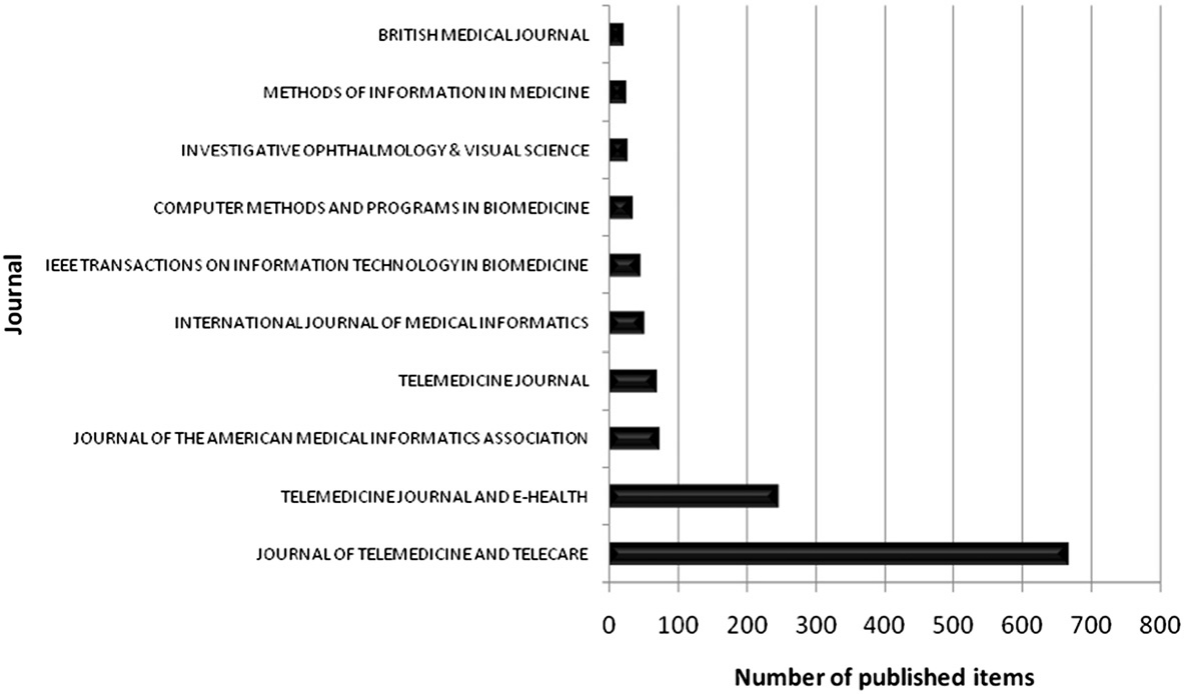
Top 10 ranking of sources by the number of published items during the period from 1900 to 2006

The ten most productive journals include three journals with the main focus telemedicine, such as “Journal of Telemedicine and Telecare”, “Telemedicine Journal and E-Health” and “Telemedicine Journal”. Another five have the main focus Information/Informatics, which are the “Journal of the American Medical Informatics Association”, the “International Journal of Medical Informatics”, the “IEEE Transactions of Information Technology in Biomedicine”, the “Computer Methods and Programs in Biomedicine” and the “Methods of Information in Medicine” (Fig. 7).

### Analysis of assigned subject categories

**Fig. 8:**
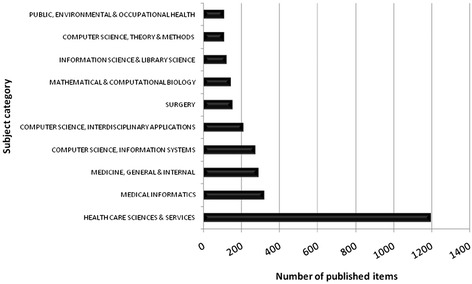
Top10 list of assigned subject categories of published items related to telemedicine; study period from 1900 to 2006

**Fig. 9:**
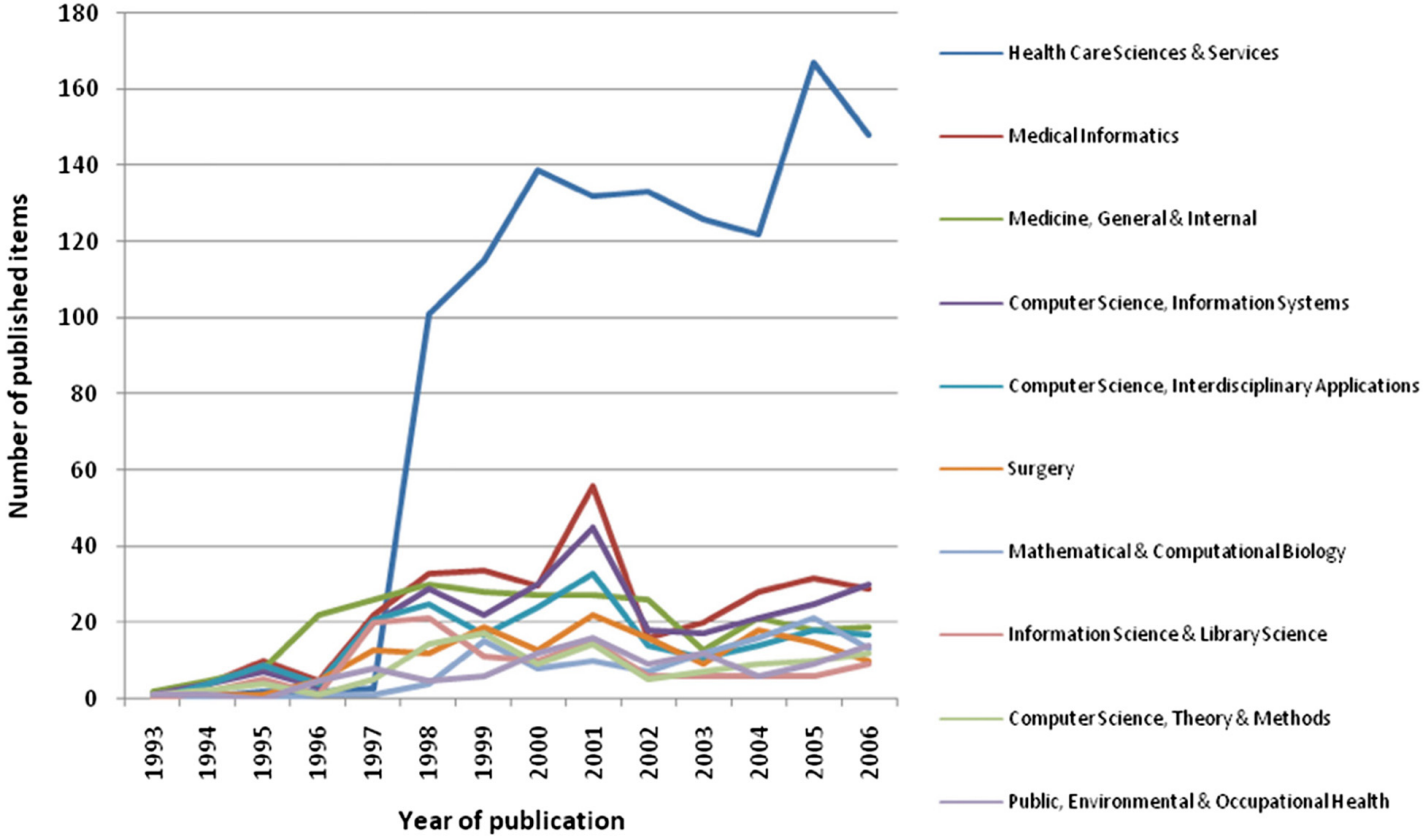
Time dynamics of top 10 assigned subject categories; study period from 1900 to 2006

In all subject categories examined for published items related to telemedicine, “Health Care Sciences & Services” ranked first by far, followed by the categories “Medical Informatics” and “Medicine, General & Internal” (Fig. 8). While the numbers of publications in most categories show a less steep growth, the category “Health Care Sciences & Services” has increased remarkably since 1997. All categories showed a temporary increasing of publication numbers in 2001 (Fig. 9).

## Discussion

The NewQIS platform decided to assess telemedicine on a scientometric level due to its increasing importance for public health and occupational and environmental medicine. Especially in countries in which the health systems are not area-wide, or occupational offshore settings, telemedical inventions may be of utmost importance. The present study provides a first precise international bibliometric evaluation of telemedicine-related research in a closed an defined time period which was set between 1900 and 2006. The methodology is based on internationally established databases, such as the Web of Science [10], and novel bibliometric tools including density-equalizing mapping [14]. The time span in some search routines was restricted to the period between 1976 and 2006. This period was chosen because the worldwide number of published items before 1976 was relatively low. Generally, there is a constant increase of interest in this field since the middle of the 1990ies. The main reason for this is the rapid progress and development in the field of communication technologies in this era. New methods of digitizing images and compressing data, as well as video coverage via low bandwidth enabled new telemedical options [15]. This strong progress in the area of computer and network technologies mainly initiated the development of telemedicine.

Data analysis of productivity parameters shows that research groups from the US maintain a leadership position in research productivity concerning telemedicine, along with Great Britain and Canada. More than 56 % of all publications are originated from these three countries.

By bringing the number of publications in relation to the respective population it can be seen that the USA with a ratio of approx. 4.3 publication/1 m residents do have a significantly lower output than the UK with 7.8 publication/1 m residents. Making the same calculation for Australia, Norway and Finland, a comparatively high ratio of approx. 10 publication/1 m for Australia, 12.4 publication/1 m for Norway and 9.2 publication/1 m for Finland can be found.

The reason for these results might be that territorial states such as Australia or Scandinavia do have special requirements regarding medical care, which accelerate the development of telemedicine in these countries [16]. Therefore, telemedicine is very advanced in those countries where medical specialists are not available in most of the regions, or where climate issues cause serious transportation issues, as is the case in wide territories of Scandinavia.

While telemedicine is highly accepted and used in countries such as Scandinavia, Australia or the USA, Germany still struggles with this new technology [17]. In Germany only 2.3 published items per 1 m residents could be found. One of the main reasons for this is the unregulated compensation situation, together with the high physician concentration. Medical professionals in Germany are still very conservative regarding telemedicine, because they are afraid of possible staff savings and quality losses caused by this new technology [18].

Whereas the number of published items was considered as an indicator for research productivity, the average citation per item was used as a sign for research quality as generally accepted. Therefore all articles were analyzed regarding the average citation of items published in each particular country. Using this average citation per item index without thresholds, Bangladesh appears to have the highest rank, followed by Iceland and Saudi Arabia. It has to be annotated that the results for those countries with a very small amount of published items appear disproportionately high. To objectify these outliers, a threshold of ten published items was introduced and, as a result, Bangladesh (1 publication), Iceland (1 publication) and Saudi Arabia (1 publication) are no longer included in the ranking. Ireland (10.19 citations/item and 80 publications) moves up to first position, followed by New Zealand and Finland (Table 2).

This result once again underlines the importance of telemedicine in territorial countries, such as New Zealand or Finland. Because of Finland being highly developed in matters of telecommunications, numerous telemedicine projects could successfully be established in this region [19]. Today telemedicine is very well implemented in the Finish health care system, so that necessary waiting time shortenings for seeing a medical specialist could be achieved [20].

When focusing on assigned categories in the Web of Science database related to telemedicine, the field “Health Care Sciences & Services“plays a leading role, with a steep increase of published items since 1997. More than 36 % of all publications are assigned to this category, followed by “Medical Informatics” with 9.72 % and “Medicine, General & Internal” with 8.84 % of all publications. This trend confirms the high impact of telemedicine for the health care system. Telemedicine is no longer only a vision which is covered in theoretical or technical fields, such as “Computer Science, Theory & Methods” or “Computer Science, Information Systems”. Today telemedicine is reality and successfully applied in a high number of medical fields [21]. Hence, more and more publications deal with the application of telemedicine in order to optimize the health care system. It is also an interesting aspect that the number of publications in the category “Health Care Sciences & Services” was insignificantly low till the mid 1990ies and then abruptly took a turn to become the most important field (Fig. 9). This change can be explained with the improvement of telecommunication technologies in that time, which eventually enabled the development of telemedicine.

## Conclusion

The present study represents the first detailed scientometric analysis of the research regarding telemedicine. The data shows clearly a strong increase in research productivity. Using science citation analysis it can be assumed that there is a large increase of interest in telemedicine studies. While the majority of data originates from the USA, smaller countries such as Ireland and New Zealand take a lead in citation per item rankings. Future studies should address the development of the field after 2006.
